# Techniques to Control Microbial Contaminants in Nonsterile Microalgae Cultivation

**DOI:** 10.1007/s12010-020-03414-7

**Published:** 2020-08-18

**Authors:** Daniel Pleissner, Astrid Victoria Lindner, Ranga Rao Ambati

**Affiliations:** 1 Sustainable Chemistry (Resource Efficiency), Institute of Sustainable and Environmental Chemistry, Leuphana University of Lüneburg, Universitätsallee 1, C13, 21335 Luneburg, Germany; 2 Institute for Food and Environmental Research e. V., Papendorfer Weg 3, 14806 Bad Belzig, Germany; 3 Center of Excellence, Department of Biotechnology, Vignan’s Foundation for Science, Technology and Research (Deemed to be University), Vadlamu, Guntur, Andhra Pradesh di-522316 India

**Keywords:** Contamination, Xenic conditions, Heterotrophy, Phototrophic, Bioeconomy

## Abstract

The aim of this mini-review with own results was an identification of techniques to suppress the growth of microbial contaminants under photo- and mixotrophic conditions. Techniques identified are the modification of environmental conditions, such as pH, oxygen, and nutrient concentrations, as well as the application of pulsed electric field, ultrasonication, and surfactants. In phototrophic cultivations, the mentioned techniques result in a decrease of number of predatory cells, but not in a complete removal. Measures to suppress the growth of contaminations (e.g., bacteria and fungi) in mixotrophic cultivations could not be identified. The co-cultivation of algae and fungi, however, was found to be beneficial for the utilization of unusual carbon compounds (e.g., phenolic compounds).

## Introduction

Microalgal biomass allows the formation of various products of low (bioenergy) and high value (pharmaceuticals) [1, 2]. The ability of microalgae to use carbon dioxide and sunlight (phototrophy), organic waste streams (heterotrophy), or both (mixotrophy) [3] has been leading to the development of cultivation systems within the frame of bioeconomy.

Due to light inhibition and light saturation, biomass concentration obtainable in phototrophic cultivation systems, such as open ponds and closed tubular systems, is rather limited [4]. Contrarily, using heterotrophic cultivation, which relies on organic carbon sources, up to 100 times higher biomass concentration can be achieved [5]. Even though pure and expensive organic nutrients, such as glucose and amino acids, were successfully substituted by hydrolysates of organic waste streams [6–8], sterilization of substrate and equipment is a major economic drawback. Sterilization, however, is necessary since bacteria with faster growth rates can outcompete algal strains [9]. In phototrophic cultivation systems, where the concentration of free organic carbon sources is negligible, the presence of bacteria is rather unproblematic. The cultivation of phototrophic algal cells, however, is threatened by the appearance of predators feeding on cells. *Colpoda steinii*, for instance, can diminish a dense culture of the cyanobacterium *Synechocystis* sp. in a relatively short time [10].

The appearance of predators has raised the question for techniques to avoid or inhibit the growth of microbial contaminations. A known measure is the modification of environmental conditions towards non-favorable conditions for a certain contamination, but less affecting the cultivation of the wanted algal strain [10–12]. Contrarily to the jeopardies associated with contaminations, the co-cultivation of algae and bacteria and/or fungi and the use of their biochemical properties can increase the substrate availability or product portfolio. However, question has been raised on whether it is possible to perform a co-cultivation under controlled conditions.

This mini-review with own results investigates techniques of nonsterile microalgae cultivation. After presenting phototrophic and mixotrophic cultivations carried out under nonsterile conditions, benefits of co-cultivation (e.g., improved productivity (13)) and techniques to control contamination will be reviewed. Eventually a synthesis will be drawn in order to critically assess the possibility of nonsterile photo- and mixotrophic cultivations.

## Materials and Methods

### Algae Cultures

#### Phototrophic Cultivation

*Chlorella vulgaris* (SAG 211-19) was obtained from the Culture Collection of Algae at Gottingen, Germany, and cultivated in 300-mL glass tubes containing BG11 medium at room temperature (25 ± 3 °C), a light intensity of 300 μmol m^−2^ s^−1^, and a pH of 7 to 8.

Three repeated cultivations were performed in a 240-L flat plate photobioreactor (1.2 × 2.4 × 0.1 m, height × length × thickness) in a greenhouse environment using BG11 medium. The top opening of the photobioreactor was covered with a glass plate. Temperature and pH were measured twice a day and maintained at 28 ± 2 °C and between pH 7 and 9 by carbon dioxide injections. Mixing and carbon dioxide injections were carried out by compressed air enriched with 1–2% (v/v) carbon dioxide at a flow rate of 50 mL min^−1^.

#### Mixotrophic Cultivation

*C. vulgaris* (SAG 211-19) growth experiments were conducted in an incubator at 30 °C (Aqualytic TC 135 S incubator, Phoenix RS-OS 20 shaker, two Voltolux LED lamps, 5 W), with a light intensity of 50 μmol m^−2^ s^−1^ and AF6 medium or olive mill wastewater (OMW) diluted to 1, 6, and 12% (v/v) with AF6 medium. OMW was obtained from an olive oil production site in Burhaniye, Turkey (Duzen Biological Sciences Research, Development and Production Co.). In this study, fresh OMW was used with a total phenolic content of 7 g L^−1^, a total phosphorus concentration of around 0.1 g L^−1^, and total nitrogen content of around 0.9 g L^−1^. OMW was pre-treated by centrifugation, filtration, and autoclavation at 121 °C for 20 min. Despite of autoclavation, it contained spores of an unknown fungus.

Cultivation occurred in 250-mL Erlenmeyer flasks with a working volume of 100 mL and a starting optical density of 0.1 and shaken at 130 rpm. The pH of the medium was set to 6.8 prior cultivation, however, by adding the OMW the pH dropped accordingly (pH of 6.0 with 1% OMW down to 5.0 with 12% OMW). The duration of the experiment was set to 10 days, and daily samples were taken under sterile conditions.

### Growth Determination

In phototrophic cultivations *C. vulgaris* cells were counted using a hemocytometer (Hausser Scientific, Horsham, PA, USA). In mixotrophic cultivation, the growth of *C. vulgaris* was monitored by measuring the optical density at 750 nm using a spectrophotometer (Macherey-Nagel, Nanocolor UV/Vis II).

### Folin-Ciocalteu Assay

For the determination of total phenolic compounds in OMW, the Folin-Ciocalteu (FC) assay was applied. To demineralized 1.8-mL water in a 10-mL glass tube, 0.2-mL OMW and 0.2-mL FC reagent were added. After stirring well on a vortexer, the mixture was placed in the dark at room temperature for 5 min. Hereafter, 2 mL of a 7% (w/v) aqueous Na_2_CO_3_ solution were added for neutralization and development of the typical blue color, as well as a 0.8 mL demineralized water to reach a 5 mL total volume. The mixture was vortexed again and kept for an incubation time of 90 min in the dark; subsequently the mixture was measured spectrophotometrically at 750 nm. The concentration of total phenolic compounds was determined by using a tyrosol calibration curve.

## Predators in Phototrophic Cultures

**Fig. 1:**
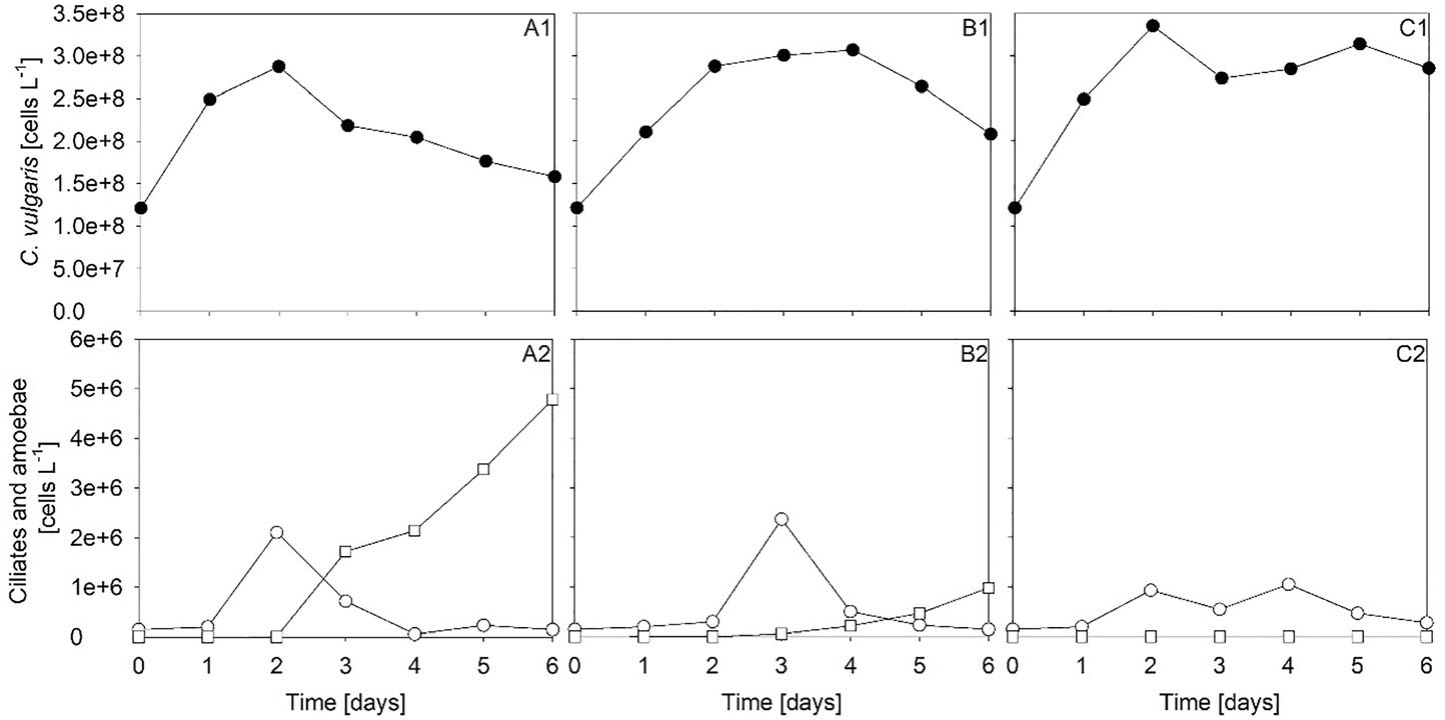
Repeated cultivation of *C. vulgaris* under phototrophic conditions*.* Cell number of *C. vulgaris* (A1, B1, and C1) and numbers of amoebae (open square) and ciliates (open circle, A2, B2, and C2) appearing in each repeated cultivation, respectively

In own phototrophic *C. vulgaris* cultivations, predators in the form of ciliates and amoebae were found to appear (Fig. 1), while the number of cells of *C. vulgaris* decreased. There was no clear trend between the three repeated cultivations regarding the appearance of predators. In the cultivation shown in Fig. 1A1 and A2, a six times higher cell number of ciliates was found compared with the cultivations shown in Fig. 1B2 and C2. Amoebae developed quickly after 2–3 days and almost disappeared again after 4 days. Predator species found were the amoebae *Platyamoeba* sp., *Vannella* sp., and *Acanthamoeba* sp., and the ciliate *Colpoda* sp.

During cultivation, pH and temperature steadily increased to 9 and 30 °C, respectively. Such a change in pH and temperature can alter the availability of carbon, trace metals, as well as essential nutrients and eventually change the distribution of species. It seems from Fig. 1 that a moderate growth rate of *C. vulgaris* prevented predators from growing. However, after microalgal growth stopped, the predator’s population increased steadily (Fig. 1A2) and the higher the number of predatory cells, the lower the number of *C. vulgaris* cells. The question raised was whether an appearing growth of predatory cells can be controlled by simple techniques applied to a running culture?

### Environmental and Chemical Techniques

The compromise of environmental conditions of two or more microbes results in conditions, which are not optimal to all microbes, but more unfavorable to one than to the other. The less affected strain will survive. For instance, when algal cells do grow slow, they become prey for predators (Fig. 1), which can cause a collapse of the whole culture. Cells do grow slow under nutrient deficiency and light limitation and/or when environmental conditions are unfavorable. For instance, a dense culture may undergo self-shading, which results, despite the presence of sufficient nutrients, in a reduced growth rate [11]. Flynn et al. further addressed the lack of knowledge on maximal biomass productivity under simultaneous minimization of zooplanktonic predation [11]. They followed an interesting approach by manipulating biomass composition of algal cells to be of low nutrient quality to predators. A lack of phosphorus results in more grazer-resistant cells. A lack of nitrogen results in biomass, which is distasteful to zooplankton [11]. The approach, however, might be in conflict to commercial interests and a specific wanted biomass composition.

A technique which has been widely applied to control microbial contaminations is the altering of pH. The predatory bacterium *Vampirovibrio chlorellavorus* feeds on living algae, breaches through the wall of *Chlorella* sp. cells, and digests it [14]. *V. chlorellavorus* can, if unnoticed, diminish a whole *Chlorella* sp. culture in a relatively short time. In order to overcome this risk, Ganuza et al. tested an easy treatment to protect *Chlorella* sp. from this predatory bacterium in 1000 and 130,000 L open ponds [12]. Once noticed that *V. chlorellavorus* is present, they exposed *Chlorella* sp. cells to a pH of 3.5 for 15 min in the presence of 5 g L^−1^ acetate. The pH was lowered using HCl and returned to pH 7.5 using NaOH. Acetate was added to simulate mixotrophic conditions. This fast treatment prevented from culture crash and can even be carried out preventively.

Touloupakis et al. applied another strategy in order to protect the cyanobacterium *Synechocystis* sp. from contaminants. Their results revealed that it is possible to grow *Synechocystis* sp. free of contaminants at a pH of 11 [15]. They further found a decreased carbohydrate concentration and loss in productivity but similar lipid content when grown at this pH compared with pH 7. This treatment was tested for protozoa and competitive microalgae, such as *Poterioochromas* sp. The effect against *Poterioochromas* sp. is of interest as this strain is an efficient phagotroph feeding on cyanobacterial cells and a common contamination in *Synechocystis* sp. mass culture.

When cultures are grown on carbon dioxide and at a high pH, the availability of carbon dioxide can be limiting. At a pH of 11, the HCO_3_^−^ availability is reduced to 17.6%. *Synechocystis* sp. can handle such low carbon availability. Furthermore, Peng et al. used the inhibiting effect of NaHCO_3_ to control microalgae-preying protozoa in *Neochloris oleoabundans* cultures (16). The growth of *N. oleoabundans* was not affected when 160 or 220 mM L^−1^ NaHCO_3_ was added to the medium, but protozoa were completely inhibited. The inhibiting effect relies on an overload of inorganic ions, competing against the assimilation of other inorganic ions and causing DNA laddering.

Another approach to control the growth of *Poterioochromas malhamensis* was presented by Ma et al. [17]. In their study, *P. malhamensis* was treated by a low pH (6, 6.5, 7, or 7.5) maintained by supplying different concentrations of carbon dioxide (v/v, 10–30, 6–15, 1–6, or 0.5–2%). A low pH of 6–6.5 reduced the number of protozoa cells. However, their results further revealed that it was not the pH itself, but the high concentration of carbon dioxide_._ The authors expected the increased carbon dioxide concentration to cause a decrease in the intracellular pH and consequently an inhibition effect.

The ciliated protozoa *C. steinii* has been shown to clear a dense culture of the cyanobacterium *Synechocystis* sp. [10]. Measures, such as high salinity (20 g L^−1^), high pH (9), and high ammonia concentration (200 mg L^−1^ at pH 8.5), as well as carbon dioxide asphyxiation (262 mg L^−1^ dissolved), did not inhibit *C. steinii*. Partially anoxic conditions during dark period, however, did result in an inhibition. Even though the actual inhibition mechanism is not known, the authors concluded that the active, grazing cells were harmed.

Another approach was followed by Gan et al. [9]. In their study, triticonazole, a sterol 14-demethylase inhibitor, was used to suppress the growth of fungi in a *Nannochloropsis oceanica* culture. *N. oceanica* was not inhibited in growth when 5 μg mL^−1^ triticonazole was applied. Fungi, however, were affected and their growth inhibited. Their results further revealed that triticonazole has different effects on algal species. Contrarily to *N. oceanica*, *Phaeodactylum tricornutum* was inhibited in growth when exposed to triticonazole. The authors further found that different sterol biosynthetic inhibitors have different effects and contrarily to triticonazole, tebuconazole inhibited the growth of *N. oceanica*. The authors concluded that the application of sterol biosynthetic inhibitors can not only control fungal growth but also the growth of unwanted algal species in mass cultivations.

It needs to be admitted that the control of contaminations using environmental or chemical measures may possibly also limit growth of algae. The known measures are species specific and more research is needed to identify measures that work species unspecific.

### Physical Techniques

Environmental and chemical techniques, such as nutrient availability and altering of pH, will besides targeting organisms also affect the wanted organism. Thus, also physical techniques in controlling microbial contaminants can be considered. For instance, the protozoan contamination in industrial scale microalgae photobioreactor can be controlled by pulsed electric field (PEF) (18). PEF causes a permeabilization of the biological cell membrane and depends on transmembrane potential of the cell membrane, where a specific transmembrane voltage threshold exists. Selecting the right strength and duration can result in the electroporation of the membrane. Rego et al. treated a *Chlorella* sp. culture containing rotifers for 6 h with typical pulses of 5 kV and 100 A with 50 Hz and 65 μs pulse width [4]. The average pulse energy was 2 × 16.25 J per pulse. After 6 h of treatment, rotifers showed an external damage of membranes. The population of rotifers decreased substantially, while *Chlorella* sp. increased in growth rate.

Another approach was presented by Wang et al. who used ultrasonication with the power of 495 W at 100% amplitude and a treatment frequency of 1 h per day [19]. This treatment was proven to control the contamination of *Poterioochromas* sp. in a 60-L *C. vulgaris* culture. *Poterioochromas* sp. was destroyed by cell wall disruption.

## Microbial Contaminations in Mixotrophic Cultures

**Fig. 2:**
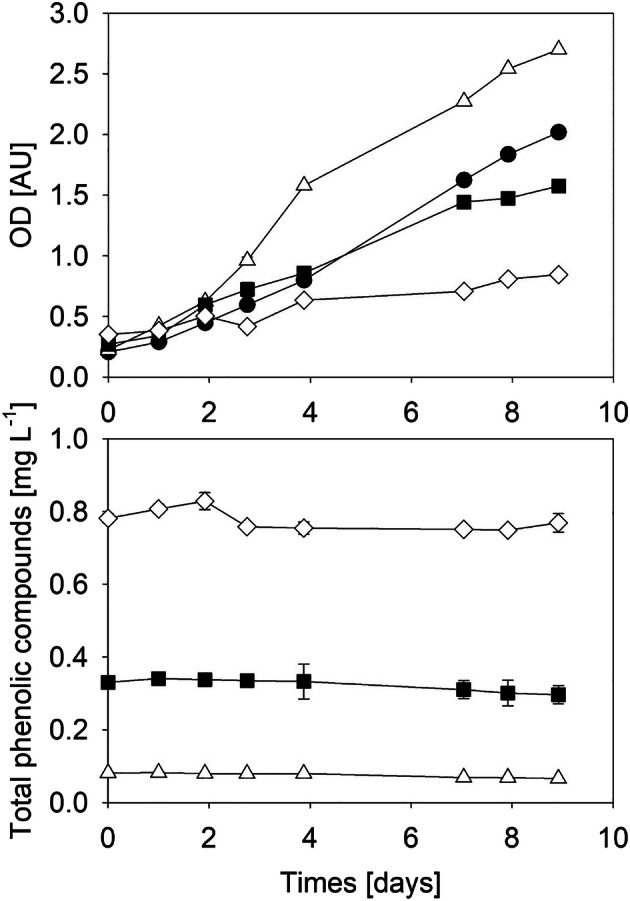
Cultivation of *C. vulgaris* under mixotrophic conditions in control medium and OMW in the presence of a fungal contamination. Optical density (OD) and total phenolic compounds were determined when *C. vulgaris* was grown in control medium (closed circle), 1% (v/v) OMW (open triangle), 6% (v/v) OMW (closed square), or 12% (v/v) OMW (open diamond)

In cultivations where organic carbon sources are supplied, bacteria and/or fungi find beneficial conditions to develop. A growing contamination is generally problematic as process productivity and efficiency are reduced. However, the presence of another organism can contribute to the detoxification and metabolization of compounds. In Fig. 2, the cultivation of *C. vulgaris* in AF6 medium and diluted OMW (1, 6, and 12%, v/v) is shown. In all cultivations, a not classified fungal strain appeared at day 3. The fungal strain did not affect the growth of *C. vulgaris* but supported the removal of phenolic compounds. Almost 20 or 10% of the total phenolic compounds were removed when 1 or 6% (v/v) OMW was applied.

Phenolic compounds in OMW can inhibit the growth of algal strains [20]. However, in Fig. 2, it is shown that the highest optical density was obtained in the presence of 1% (v/v) OMW. The lowest optical density was found in 12% (v/v) olive mill wastewater where the high concentration of phenolic compounds inhibited the growth of *C. vulgaris*.

Despite the present contamination, *C. vulgaris* was the predominant species, and the fungal contamination did not significantly affect optical density. The partly removal of phenolic compounds in 1% (v/v) may have contributed to the growth of *C. vulgaris*.

It was found that bacteria and microalgae interacted with each other when they are co-cultured (21), and thus interactions can also have beneficial effects on productivity and efficiency of the cultivation of algae. Heterotrophic organisms, for instance, provide carbon dioxide to photoautotrophic algae, which in reverse supply oxygen [22]. *Scenedesmus* sp. produces a range of low molecular compounds, such as lactic, oxalic, hydroxybutyric, and succinic acids, glycerol, indole, and pyrrole derivatives as well as some organic phosphates and N-alkyl glycine derivatives, which may suppress the growth of bacteria [23]. The challenge lies in the construction of consortia with synergistic effects. It has been shown in *P. tricornutum* cultures that such a consortium can be created [24] and the organisms, associated with a consortium, depend on the function to be carried out by the bacteria and the environmental conditions.

Berthold et al. presented an approach where *Characium* sp. was co-cultivated with *Pseudomonas composti* [25]. The authors inoculated the culture with a certain number of cells of *P. composti*. *Characium* sp. is a mixotrophic alga, and thus it was assumed that it benefits of the compounds released by *P. composti*. Furthermore, the effect of cell-free filtrate on the performance of *Characium* sp. was investigated. The study revealed improved biomass yield and lipid content of *Characium* sp. when cell-free extracts or intact cells were added. For instance, the biomass yield increased by 42.3%, compared with control when *P. composti* was added. The authors concluded that this effect may result from the frequent association of the phylum *Pseudomonas* with green algae and the stimulating effect of released compounds on *Characium* sp. The identity of the released compounds, however, remains unclear. It should also be mentioned that Berthold et al. investigated 39 bacterial strains and found only a synergistic effect when *Characium* sp. and *P. composti* were co-cultivated [25]. Thus, co-cultivation seems a promising approach, but intensive screening is necessary.

Cho et al. used an ecologically engineered bacterial consortium to enhance microbial biomass and lipid productivities [26]. They used xenic cultures of *C. vulgaris* and *Scenedesmus* sp. and identified the associated bacteria by sequencing 16S rRNA. They found a broad range of associated bacteria and selected four dominant bacterial strains, such as *Flavobacterium*, *Hyphomonas*, *Rhizobium*, and *Sphingomonas*, along with two least dominant strains, *Microbacterium* and *Exophiala*. The least dominant strains were found to have a negative effect. *Microbacterium* competed for essential nutrients and *Exophiala* was algacidal. The four dominant species were beneficial to the growth. The beneficial effect originates from the bacterial utilization of organic carbon released by microalgae. Bacteria, in return, supply inorganic compounds and low-molecular-weight organic carbon. Grotkjaer et al. have shown the effect of the probiotic bacteria *Phaeobacter inhibens* in *Tetraselmis suerica* cultures [27]. They found that *P. inhibens* is able to antagonize *Vibrio anguillarum* in live feed systems, which was concluded as promising, as it further develops the probiotic concept.

## Synthesis

The abovementioned techniques and measures illustrate options to deal with contaminations in microalgae cultures. It is, however, in most cases trial and error to find the best option for a certain contamination. Particularly the controlled co-cultivation of algae and bacteria and/or fungi, in order to establish beneficial effects as shown above or listed by Lian et al., requires intensive investigation to design an appropriate consortium [13]. Even though it is known that certain microalgae live in a symbiosis with, for instance, bacteria, rather little information is available on how such a symbiosis works. It remains unclear whether it is an exchange of nutrients [25, 26] or essential compounds, such as vitamins [28], passive signaling supported by the excretion of molecules [29], fixation of nitrogen [30] or providing of nitrogen compounds [31], modulation of excreted compounds by bacteria [32], sharing of iron and/or fixed carbon [33], mutualistic interaction [34], or both mutualistic and commensalism interactions [35]. Furthermore, the interaction of algae and other organisms depends on nutrient availability and environmental conditions, and even though algae live in a symbiosis with other organisms, they are not protected for being taken over by those [32].

Another aspect that needs to be considered is the competition for nutrients among different microorganisms and the effect on their growth rates in a co-cultivation. Rhee reported an inhibition of the growth of *Scenedesmus* sp. when grown in the presence of *Pseudomonas* sp. [36]. Contrarily, the growth of *Pseudomonas* sp. was not affected. However, algae show a higher accumulation of phosphate in form of polyphosphate, also known as luxury consumption, then bacteria, and this may give an advantage over fast-growing bacteria at the beginning of a cultivation when sufficient phosphate is supplied [36]. The competition for carbon and nitrogen compounds, however, cannot be solved by supplying nutrients in excess. A luxury consumption of carbon and nitrogen compounds has not been shown for algae and it is expected that the faster-growing species (bacteria and fungi) might be the one that consumes most of the nutrients.

Even for phototrophic cultivations, the application of specific techniques to control contaminations in terms of number of cells or design of consortia still need to be shown reliable for a long period of time and at industrial scale. The modification of environmental conditions, such as nutrient supply and pH, can potentially decrease the number of cells of contaminants. Apparently, protozoa are more sensitive to unfavorable conditions than algae, and thus contaminations can be instantly reduced, but not completely removed. A similar acute effect on contaminations is associated with the techniques: pulsed electric field and ultrasonication. Similarly to the modification of environmental conditions, contaminations are not completely removed, but number of cells reduced.

## Conclusions

Research projects dealing with the cultivation of microalgae for a certain purpose include mostly a screening of algal strains (either axenic cultures or xenic isolates from natural habitats, which are made axenic) to find the most promising candidate. To use beneficial effects that come along with a co-cultivation, the most promising strategy would be to simultaneously isolate algae and bacteria and other associated microorganisms. The future task might be to screen for certain effects rather than certain microorganisms.
