# Correction to: Techniques to Control Microbial Contaminants in Nonsterile Microalgae Cultivation

**DOI:** 10.1007/s12010-021-03693-8

**Published:** 2021-10-28

**Authors:** Daniel Pleissner, Astrid Victoria Lindner, Ranga Rao Ambati

**Affiliations:** 1 Sustainable Chemistry (Resource Efficiency), Institute of Sustainable and Environmental Chemistry, Leuphana University of Lüneburg, Universitätsallee 1, C13, 21335 Luneburg, Germany; 2 Institute for Food and Environmental Research E. V, Papendorfer Weg 3, 14806 Bad Belzig, Germany; 3 Center of Excellence, Department of Biotechnology, Technology and Research (Deemed To Be University), Vignan’s Foundation for Science, Andhra Pradesh di-522316, Vadlamu, Guntur, India

**Correction to: Applied Biochemistry and Biotechnology (2020) 192:1376–1385**

**https://doi.org/10.1007/s12010-020–03414-7**

The article “Techniques to Control Microbial Contaminants in Nonsterile Microalgae Cultivation,” written by Daniel Pleissner, Astrid Victoria Lindner, and Ranga Rao Ambati, was originally published Online First without Open Access. After publication in volume 192, issue 4, page 1376**–**1385, the author decided to opt for Open Choice and to make the article an Open Access publication. Therefore, the copyright of the article has been changed to © The Author(s) 2021 and the article is forthwith distributed under the terms of the Creative Commons Attribution 4.0 International License, which permits use, sharing, adaptation, distribution, and reproduction in any medium or format, as long as you give appropriate credit to the original author(s) and the source, provide a link to the Creative Commons licence, and indicate if changes were made.

The images or other third party material in this article are included in the article’s Creative Commons licence, unless indicated otherwise in a credit line to the material. If material is not included in the article’s Creative Commons licence and your intended use is not permitted by statutory regulation or exceeds the permitted use, you will need to obtain permission directly from the copyright holder.

To view a copy of this licence, visit http://creativecommons.org/licenses/by/4.0/

Open Access funding enabled and organized by Projekt DEAL.

